# Prediction of postoperative liver regeneration from clinical information using a data-led mathematical model

**DOI:** 10.1038/srep34214

**Published:** 2016-10-03

**Authors:** Kimiyo N. Yamamoto, Masatsugu Ishii, Yoshihiro Inoue, Fumitoshi Hirokawa, Ben D. MacArthur, Akira Nakamura, Hiroshi Haeno, Kazuhisa Uchiyama

**Affiliations:** 1 Departments of General and Gastroenterological Surgery, Osaka Medical College Hospital, Osaka, Japan; 2 Mathematical Biology Laboratory, Department of Biology, Faculty of Sciences, Kyushu University, Fukuoka, Japan; 3 Mathematical Sciences, University of Southampton, SO17 1BJ, UK; 4 Human Development and Health, Faculty of Medicine, University of Southampton, SO17 1BJ, UK; 5 Department of Radiation Oncology, Massachusetts General Hospital, Boston, MA, USA

## Abstract

Although the capacity of the liver to recover its size after resection has enabled extensive liver resection, post-hepatectomy liver failure remains one of the most lethal complications of liver resection. Therefore, it is clinically important to discover reliable predictive factors after resection. In this study, we established a novel mathematical framework which described post-hepatectomy liver regeneration in each patient by incorporating quantitative clinical data. Using the model fitting to the liver volumes in series of computed tomography of 123 patients, we estimated liver regeneration rates. From the estimation, we found patients were divided into two groups: i) patients restored the liver to its original size (Group 1, n = 99); and ii) patients experienced a significant reduction in size (Group 2, n = 24). From discriminant analysis in 103 patients with full clinical variables, the prognosis of patients in terms of liver recovery was successfully predicted in 85–90% of patients. We further validated the accuracy of our model prediction using a validation cohort (prediction = 84–87%, n = 39). Our interdisciplinary approach provides qualitative and quantitative insights into the dynamics of liver regeneration. A key strength is to provide better prediction in patients who had been judged as acceptable for resection by current pragmatic criteria.

Although the capacity of the liver to recover its size after resection has enabled extensive liver resection, liver resection is fraught with risk and the potential for complications due to the complex operative interventions^1^. Post-hepatectomy liver failure (PHLF), which is defined as a post-operatively acquired deterioration in the ability of the liver to maintain its normal functions, remains one of the most life-threatening complications. The incidence of PHLF varies between 1.2% and 32% in the literature^2^. Although platelet count, patient age, graft size, and presence of the middle hepatic vein influence liver regeneration following healthy living donor transplantation^3^,^4^,^5^,^6^, prognostic factors after liver resection have not been sufficiently identified for cancer patients or patients after resection^7^,^8^. Although a practical guideline demonstrated criteria for resection in 1983, clinical decision making remains complex^9^. Provision of clear criteria for selecting patients who will benefit from liver resection is thus important and urgent.

The liver generally regenerates in a highly organized fashion after surgery, involving hyperplasia of all the cell types of the liver^10^. Imaging modalities essentially help us to assess liver regeneration by demonstrating increases in liver volume. Multi-detector row computed tomography (CT) has become an essential tool for evaluating the volume of remnant liver. CT volumetry is currently routinely performed when liver resection is planned for tumors^11^,^12^. In the previous studies, temporal-series CT images provided essential information for establishing a mathematical model to elucidate growth kinetics of a focused disease^13^,^14^,^15^,^16^. When considered alongside pre- and perioperative clinical records, a large dataset of regeneration images provide a wealth of information with which to develop a comprehensive understanding of the various patterns of postoperative liver regeneration. Our study with temporal-series volumetry information will be added to a series of theoretical investigations into the dynamics of liver regeneration reported over the past decade^17^,^18^,^19^,^20^.

The present study investigated the dynamics of liver regeneration using an interdisciplinary approach. The final goals were to propose powerful criteria to identify potential clinical predictors for successful liver resection and to predict the time course of liver regeneration. The clinical dataset consisting of 157 patients contained liver volumetry at three or more time points in 123 patients in Osaka Medical College Hospital, along with a range of pre- and postoperative clinical variables. Based on a temporal series of CT images, we developed a novel mathematical model of liver regeneration and successfully estimated liver regeneration rate after resection in each patient. From the estimation of the liver regeneration rates, we found that patients were divided into two groups: i) patients restored the liver to its original size (Group 1); and ii) patients experienced a significant post-operative reduction in size (Group 2). Using the estimated regeneration rates, we successfully described the post-hepatectomy temporal course of liver regeneration in each group. Finally, from linear discriminant analysis of regeneration rates using pre- and perioperative information, we proposed a formula to prospectively predict whether a new patient is likely to be allocated to Group 1 or Group 2. Importantly, the accuracy of our model prediction was validated using a separate cohort consisting of 39 patients. In summary, this study provides a significantly increased understanding of liver regeneration thereby informing clinical decision-making for patients with malignant or other hepatic diseases, including whether to offer operations and when to offer early adjunct therapy to patients who are likely to suffer from poor liver regeneration.

## Materials and Methods

### Clinical cohorts

Between March 2009 and June 2014, a total of 157 patients (124 men, 33 women) underwent liver resection at Osaka Medical College in Japan (training cohort) (Table S1 and Fig. S1). The median age was 68 years (range, 28–88 years). Baseline characteristics of the study population are provided in Table 1. Liver volumetry at five months after operation were available in 109 patients, and three or more time points of CT scans were available in 123 patients out of 157 patients, respectively (Table S1 and Fig. S1). On admission, laboratory results for aspartate aminotransferase (AST), alanine aminotransferase (ALT), body mass index (BMI), diabetes mellitus, total bilirubin, albumin, prothrombin time, platelet count, and hepatitis B virus (HBV) and hepatitis C virus (HCV) were routinely collected. The indocyanine green retention rate at 15 min (ICGR15) was examined in 103 patients (Table S1 and Fig. S1). A validation cohort consists of 39 patients who underwent liver resection at Osaka Medical College in Japan between June 2014 and May 2016 (Tables S2-S3 and Fig. S1). Full clinical variables including ICGR15 and three or more time points volumetry were available in all patients (Table S2). Data collection and analysis were approved by the Ethics Committee on Clinical Investigation of Osaka Medical College Hospital (Nos 1029 and 1198) and all patients were fully informed of the study design according to provided written, informed consent to participate in this study. Methods were carried out in accordance with the approved guidelines.

### Measurement of liver volume

For the calculation of total liver volume, commercial interactive volumetry-assist software (SYNAPSE VINCENT; Fujifilm, Tokyo, Japan) was used. Volumetric measurements were performed as previously reported^21^. Major vessels, including the vena cava and extrahepatic portal vein, as well as major fissures, such as the fissure for the ligamentum teres, were excluded by hand tracing of organs. The circumscribed areas were then automatically multiplied by the CT section thickness of 5 mm, yielding an approximate value for the liver volume. Liver volumes were determined preoperatively and at 1 day, 7 days, and 1, 2, and 5 months and 1 and 2 years postoperatively.

### Surgical procedure and perioperative factors

The criteria for hepatic resection and the details of the surgical technique have been described in previous studies^22^,^23^,^24^,^25^. Surgical factors assessed included surgical duration, intraoperative blood loss, and resected liver volume. Postoperative complications assessed included ascites and biliary complications, which were defined according to the Clavien-Dindo classification of surgical complications^26^,^27^.

### Statistical analysis

Nonlinear least-squares curve fitting was performed to estimate the liver regeneration rate *r* in each of the 123 and 39 patients in a training and a validation cohorts, respectively. Associations between two continuous variables were tested using the Mann-Whitney U test. Categorical variables of sex (male/female), diabetes mellitus (yes/no), Child-Pugh classification (A/B), and HBV and HCV infection status (yes/no), the presence of ascites, and biliary complication were converted into dichotomized variables (one or zero, respectively). Uni- and multivariate linear regressions were performed to test the statistical significance of clinicopathological factors on determining regeneration rate *r*. Pearson’s product-moment correlation was performed to calculate the correlation between paired variables. Factors with Pearson’s correlation coefficient over 0.8 were defined as correlated. Linear discriminant regression analysis was performed to distinguish two classes of liver regeneration. Leave one out cross validation was performed to evaluate the accuracies of predictions by linear discriminant functions in a training cohort. All values of *P* < 0.05 were considered indicative of statistical significance. Statistical analyses were performed using R version 3.1.0 software (R Foundation for Statistical Computation, Vienna, Austria).

## Results

### The basic mathematical model

**Figure 1:**
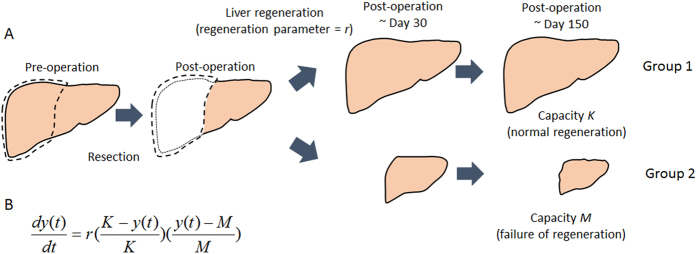
Mathematical model for liver regeneration. (**A)** Schematic diagram of the mathematical model of liver regeneration after surgical resection. The system possesses two steady states of liver volume, *K* and *M. K* and *M* represent saturated volume after liver recovery and shrunken volume after irreversible reduction in size due to liver failure, respectively. All cases will converge to one of the two states. Cases converging to *K* and *M* are denoted by Group 1 and Group 2, respectively. (**B)** A differential equation for the time change of liver volume after surgery. Liver volume and regeneration rate are denoted by 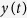 and *r*, respectively. A regeneration rate *r* in cases in which liver volume converge to *K* is estimated to be positive, while negative in a case in which liver volume converge to *M.*

To investigate the dynamics of liver regeneration, we developed a novel mathematical model that describes the temporal change in liver volume after surgery (Fig. 1). Post-operative quantitative clinical data indicated there were two major tendencies of liver regeneration: i) liver restored its original size and ii) liver reduced in size. From the aspects of the bi-stable regeneration kinetics of liver, we formulated a system with two non-zero equilibrium states. We extended a logistic function which has one non-zero equilibrium state by adding another equilibrium state. In the model, resected liver volume converges to either of two states, *K* and *M*: *K* represents the original volume of the liver in each patient before surgery, while *M* represents the reduced volume. Then, the mathematical model is given by


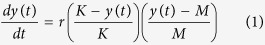


Here, *y(t)* denotes liver volume after surgical resection and *r* denotes the regeneration rate per day. For any initial volume *y*_*0*_ between *K* and *M*, the dynamics in the model will converge to one of these two states. The analytical solution to Eq. (1) is given by


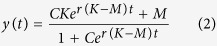


Here, *C* is given by 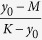. See supplementary information S1 for details of the derivation. Please note that (i) the initial liver volume after surgery, *y*_*0*_, was standardized to 1000 in order to compare the dynamics of liver regeneration between patients and (ii) initial volume *y*_*0*_ is between *K* and *M* in any patients.

**Figure 2:**
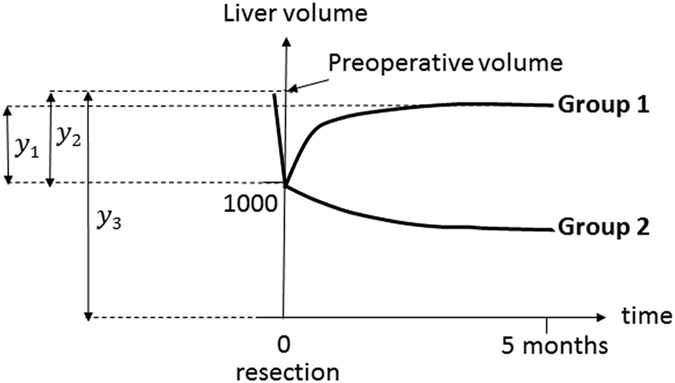
Time course of liver regeneration. Typical time course of liver regeneration. Time zero represents the time of resection. The regenerated volume at 5 months after surgery, resected volume, and original (preoperative) liver volume are denoted by *y*_1_, *y*_2_, and *y*_3_, respectively. Resection level is denoted by 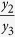. For each patient, the initial volume after surgery was standardized to 1000. *K* is defined as preoperative liver volume (=*y*_3_). Patients who fulfill requirements: (i) volume at 5 months after surgery <0.9y_3_ and (ii) y_1_<0 are used in *M* estimation (n = 15).

To apply this model to clinical classification, we determined *K* and *M* in each patient depending on the clinical data. *K* was determined as the original (preoperative) liver volume in each patient by definition (Fig. 2). To determine *M* for each patient, we investigated liver volume at five months after resection because volume information was available in more patients than at other post-operative time points and the outcome of liver regeneration became clear at that time (Table S1). Among 109 patients whose liver volume at five months after operation were available, 15 patients showed regeneration less than 90% of preoperative volume (<0.9*y*_3_) at the time and decreased in volume after surgery (*y*_1_ < 0) (Figs 2 and S1). We investigated how their liver volumes at five months after operation depended on their clinical variables, and observed correlations with preoperative liver volume and intraoperative blood loss (*P* = 0.04 and *P* = 0.06, respectively, Tables S4–S6). Therefore, we defined *M* for each patient as


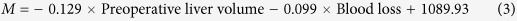


We then estimated regeneration rate *r* using temporal-series CT images at one preoperative and a maximum of seven postoperative time points in each case based on the Equation (2). A regeneration rate *r* in cases in which liver volume converge to *K* is estimated to be positive, while *r* is estimated to be negative in a case in which liver volume converge to *M* (Fig. 1).

### Estimation of regeneration rate

**Figure 3:**
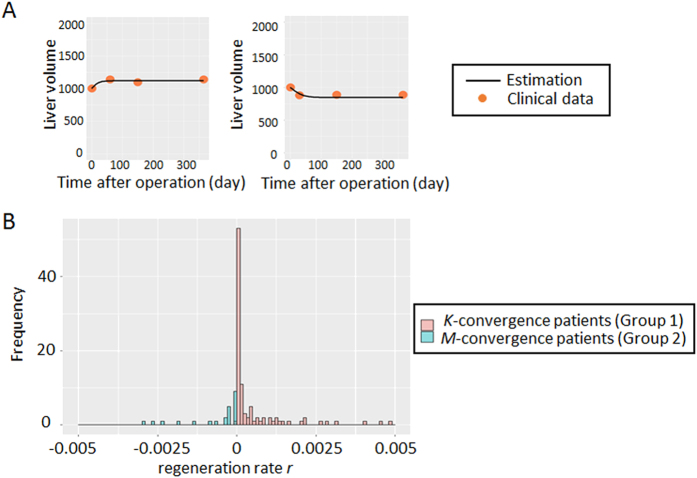
Estimation of liver regeneration rate *r*. (**A)** Representatives of non-linear least-squares curve-fitting in liver recovery using the temporal course of liver volumetry data. (**B)** Distribution of regeneration rate *r* in patients in accordance with the two classes of liver regeneration: *K* or *M*. Red bars represents cases converging to *K* (Group 1), while blue bars represent cases converging to *M* (Group 2).

We utilized our novel mathematical framework to estimate regeneration rate *r* using temporal-series CT images at one preoperative and a maximum of seven postoperative time points. By fitting Eq. (2) to the clinical data with the nonlinear least-squares method, we estimated regeneration rate *r* in each of 123 patients who had three or more time points volumetry in a training cohort (Fig. S1). Curve-fitting from two representative patients is shown in Fig. 3A. Whether a patient (i) showed regeneration of the liver to the original size or (ii) experienced a reduction in size (i.e. whether a patient was estimated to show convergence to final liver volume *K* or *M* by curve fitting) was also recorded. Preoperative liver volume, resection level, and blood loss showed the best correlations with estimated regeneration rate *r* with statistical significance (*P* = 0.0011, *P* = 0.0012, and *P* < 0.001, respectively) (Tables S7–S9).

### Liver regeneration dynamics in the two subsets of patients

**Figure 4:**
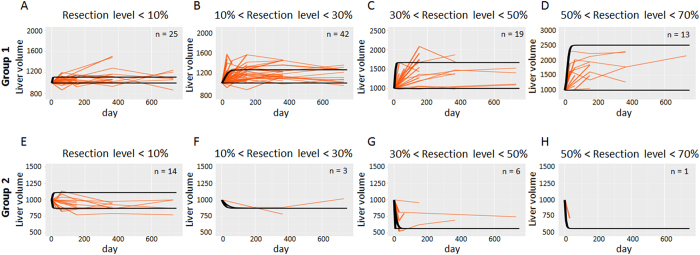
Prediction of liver regeneration dynamics. Dynamics of liver regeneration with estimated regeneration and resection levels. Cases are subdivided into two groups: those in whom liver regained its original size (Group 1) (panels A–D); and those who experienced a reduction in liver size (Group 2) (panels E–H). Both groups are then categorized into four subgroups according to resection levels, 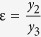 (Fig. 2): i) ε<0.1; ii) 0.1 ≤ ε < 0.3; iii) 0.3 ≤ ε < 0.5; and iv) 0.5 ≤ ε < 0.7. Here, *y*_2_ and *y*_3_ represent resected volume and original volume (Fig. 2). Two black curves in each panel represent prediction curves of liver regeneration from Eq. (2) using regeneration rates that cut off the upper and lower 10% of regeneration rates from the normal distributions shown in Figure S1. Brown curves represent clinical data of liver regeneration from 123 patients.

We investigated the dynamics of liver regeneration in the two groups that converged to *K* or *M*. Of the 123 patients with liver resection, 99 were estimated to converge to *K* (Group 1) and 24 were estimated to converge to *M* (Group 2). We investigated the distribution of regeneration rates in the two groups (Fig. 3B). Then, we predicted the time change in liver volume for the two groups (Fig. 4). To do so we considered four subgroups according to resection levels, 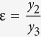, : i) ε < 0.1; ii) 0.1 ≤ ε < 0.3; iii) 0.3 ≤ ε < 0.5; and iv) 0.5 ≤ ε < 0.7 (Fig. 2). We fitted the estimated regeneration rates in each subgroup to normal distributions (Fig. S2). Then, we employed two regeneration rates that cut off lower and upper 10% of each normal distribution and drew two curves of liver regeneration with Eq. (2), so that 80% of cases would be covered between the two curves (Fig. 4). The 80% prediction intervals succeeded in covering 86.8% and 79.2% of patients converging to *K* and *M*, respectively (Fig. 4). Favorable liver regeneration was predicted in cases converging to *K* (Group 1) (Fig. 4A–D). By contrast, we observed that the liver tended to decrease in size if more than 10% was resected in cases converging to *M* (Group 2) (Fig. 4E–H). Note that simple estimation of the convergence by using only the initial time point did not predict a long period dynamics of liver regeneration. Sixteen of 99 patients who initially experienced decrease in liver volumes eventually recovered their liver volume and seven of 24 patients who showed favorable regeneration at early period converged to liver failure (Table S10 and Fig. S3).

### Threshold of liver regeneration

To identify potential candidates for liver regeneration, we finally investigated whether the regeneration rate *r* estimated by our mathematical framework, and therefore the likelihood of successful liver regeneration subsequent to resection, could be predicted from independent clinical variables. To do this, we performed linear discriminant analysis of regeneration rate *r* using preoperative clinical factors. Linear discriminant analysis finds a linear combination of prognostic factors that best separates two classes in the group of patients. The 103 patients with full clinical information available were employed to determine the discriminant function *D*_*1*_, given by


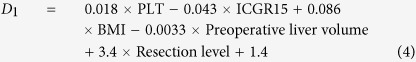


If *D*_*1*_ is positive, the volume of resected liver is predicted to converge to *K* (i.e successful postoperative regeneration) while if *D*_*1*_ is negative, the resected liver is predicted to converge to *M* (i.e poor postoperative regeneration). Using this model, 86.4% of cases were correctly classified, and so were 85.4% of cases with cross validation (Table 2).

In addition, we conducted a similar analysis using pre- and perioperative clinical factors (Table 2). In this case, the discriminant function *D*_*2*_ was given by


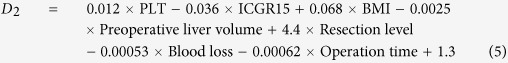


The overall predictive accuracy of classification by Eq. (5) was 89.3% and 87.4% without and with cross validation (Table 2). We finally investigated the prediction accuracy of our model by using a validation cohort (Tables S2, S3, and Fig. S1). We confirmed 84.6% and 87.2% of patients were correctly classified by using Equations (4) and (5), respectively (Table 3). Note that these equations were generated using clinical variables from previously treated 103 patients.

## Discussion

The main concern in clinical decision-making for management of patients with insufficient liver function involves whether the liver will maintain its size and function after surgical resection. In this regard, the investigation of liver regeneration dynamics using factors routinely tested in the clinic represents an important issue in practical clinical settings. This study adopted a combined clinical and theoretical approach for the goals of identifying successful/unsuccessful candidates for liver resection and monitoring post-hepatectomy liver regeneration. This translational approach was achieved by establishing a novel mathematical framework with two carrying capacities of successful/unsuccessful liver regeneration. We first obtained numerical estimates of liver regeneration in each patient based on the mathematical model and CT image data. We confirmed that the model represented the temporal course of liver regeneration in both subgroups. We then predicted whether patients would experience successful/unsuccessful regeneration by conducting discriminant analysis. Finally, we confirmed the prediction accuracy of our model by using a validation cohort. Because all the factors used in the analyses are routinely recorded in clinic settings, our model is routinely clinically applicable and is therefore directly testable in clinical trials.

This study demonstrated a method to predict whether the liver of a patient will succeed in recovering, with accuracies as high as 84–88% in both of a training cohort with cross validation and a separate validation cohort by utilizing mathematical tools (Eqs (4) and (5), Tables 2 and 3). As a number of studies addressed, the major concern in PHLF is to be a predominant cause of hepatectomy-related mortality^2^. In fact, when we investigated on patients with hepatobiliary primary diseases (HCC, CCC, and hepatic benign tumor, n = 76), the patients who converged to *M* (Group 2) showed worse survival outcome than those who converged to *K* (Group 1) (Fig. S4A). We tested if the discriminant equations, which were derived in order to distinguish patients in terms of the liver recovery, could classify patients in terms of survivorship. Interestingly, we succeeded in predicting significant difference in survival outcomes between the two groups when we stratified patients by including perioperative information in the discriminant Equation (5), although we did not find a significant difference by Equation (4) (Fig. S4B,C). By contrast to hepatobiliary primary disease, all patients with metastatic disease (*n* = 27) converged to *K* (Group 1). The discriminant Equations (4) and (5) perfectly classified patients with metastatic disease in *K* (Fig. S5). This may occur due to the baseline normal liver function in metastatic disease. These results indicate that our method is able to reliably identify patients who are likely to experience unsuccessful liver regeneration and develop life-threatening post-hepatectomy complications.

To aid understanding of our method the schematic in Supplementary Figure 6 summarizes our method. Critically, if the solution of the discriminant equation is positive, then we predict with good accuracy that the liver of the patient is likely to recover to the original size (80 of 93 *K*-predicted patients with cross validation) (Tables 2 and 3). In this case, surgery can be planned by considering the greatest benefit to the patient. However, if the solution is negative, then the patient is likely to experience poor liver regeneration (8 of 10 *M*-predicted patients with cross validation) (Tables 2 and 3). In this situation, avoiding resection is one treatment option; alternatively, minimally invasive procedures, which reduce operation time, intraoperative blood loss and the risk of postoperative complications^28^,^29^ (perioperative factors which contribute to liver recovery and therefore survival, see Eq. (5)), should be considered. Indeed, by including perioperative information in the discriminant equation, more precise patient stratification could be obtained (Tables 2 and 3). In this case, we predicted with good accuracy that 81 of 93 *K*-predicted patients showed recovery to the original size; and 9 of 10 *M*-predicted displayed unfavorable liver regeneration (Tables 2 and 3). We accurately predicted the subsequent poor survival outcomes of patients with hepatobiliary primary cancer who experienced a significant post-operative reduction in size (Fig. S4C). Since all patients enrolled in this study had once been judged as acceptable for resection by clinicians according to the current pragmatic criteria, a key strength of our model is the ability to provide better prediction even in this ‘acceptable’ cohort. Therefore our method provides more refined criteria for prediction of the success of postoperative liver regeneration, and particularly allows identification of patients who are at a greater risk of liver failure and subsequent low survivorship following surgery.

Another important clinical implication of this study is that we provide the quantification of the dynamics of liver regeneration so that we can predict the temporal course of liver regeneration in categorized patients (i.e., estimated regeneration rate *r*) (Fig. 4). Our model successfully reproduced several regeneration modes presented in a recent study^20^. From their classification, patients may experience delayed recovery or unchanged liver volume after surgery. When a regeneration rate *r* in our model is large, a rapid regeneration after surgery is expected, whereas a delayed regeneration is expected when it is small (Fig. S7). We recognized the profiles of a subset of patients did not match the two distinct steady states but represented unresponsive regeneration. To see whether these cases affect the prediction accuracy, we performed a linear discriminant analysis by omitting cases in which all time points of liver volume after resection were within 1000 ± 100 (the number of omitted cases was 14 out of 103 patients). The total predictive accuracy changed from 85.4% and 87.4% with pre- and perioperative factors with cross validation, respectively, to 85.4% and 88.8% with pre- and perioperative factors with cross validation, which indicated the predictive accuracies were comparable (Table S11). We therefore concluded that our model using the clinical patient-specific value of *r* had a predictive capacity for heterogeneous patient populations with various type of regeneration modes. Since we assume that *K* is fixed as the original liver volume, our model assumption does not include enhanced regeneration. This can be addressed in future studies. Our results also theoretically support previous findings that a rapid liver regeneration during the first month and small changes in liver volume after the first month is expected when a patient has a functional liver (Figs 4 and S7)^21^,^30^,^31^. In practical settings, clinicians can select potential candidates who will need close follow-up or adjunctive therapy early by identifying those who do not reach the predicted regeneration curve.

We used liver regeneration rates estimated by using their overall temporal-series CT images, but not their initial measurements postoperatively, to determine whether patients’ trajectories further proceeded in the positive or negative direction. This was necessary because postoperative liver volume in an early postoperative period did not always predict their prognosis in liver volume in a longer period; 16 out of 99 patients converged to *K* and 7 out of 24 patients converged to *M* failed to be estimated accurately (Table S10 and Fig. S3). Several inherent limitations must be also considered when interpreting the findings of the present study. First, although patients in the current study were enrolled and examined prospectively under the same follow-up protocol, we needed to omit patients during the eligibility period because of the lack of several clinical factors. This would have slightly reduced the continuity of the patient population. Second, recent studies have reported that other factors such as hepatocyte growth factor are associated with liver regeneration potency^30^,^32^,^33^. Most patients lack such data and future studies should include these factors into analyses for better predictive models. Our modestly sized cohort included a heterogeneous population under different treatment therapies according to disease, which might influence liver regeneration capability. Despite that, Equation (2) reproduces the dynamics of liver regeneration and provides better prediction in patients than the current pragmatic criteria. Finally, discriminant equations were generated retrospectively using clinical cohorts that received hepatectomy according to the current practical clinical criteria^9^. Due to the criteria, there was a limitation in the resectable liver volume; e.g. a case with high ICGR15 and low PLT levels, which indicate their impaired liver baseline function, could not receive larger liver resection in principle. Patients who could receive large resection had originally favorable liver function so that the contribution of resection level were positive in discriminant equations.

The liver maintains a delicate balance between volume loss and excess growth, based on complex molecular mechanisms^30^. A number of mechanisms for regulating liver size have been investigated, including the roles of cytokines, growth factors, matrix remodeling, and metabolic signals^10^. Recent mathematical models developed for liver regeneration have served well as predictors for estimating the effects of regeneration networks on the liver remnant^17^,^18^,^19^,^20^. However, such results are preliminary, and have yet to be translated into actual clinical benefits, because the signaling pathway frameworks were based on animal models. In this regard, our study is advantageous since it yields a prediction of liver regeneration after surgery only using commonly obtained laboratory data on admission. Here, routine testing of ICG clearance is also recommended, as a safe, dynamic tool for quantifying liver function^34^,^35^. Our simple mathematical framework is therefore highly applicable and can be directly tested in the clinic.

In conclusion, we have proposed an intuitive, universal mathematical model of liver regeneration. The interdisciplinary approach employing this mathematical framework and clinical data provides both qualitative and quantitative insights into the dynamics of liver regeneration. As the number in the cohort increases, we expect our methodology will provide more accurate predictions and monitoring tools for post-hepatectomy liver regeneration, allowing precise tailoring treatment regimens to individual patients.

## Additional Information

**How to cite this article**: Yamamoto, K. N. *et al.* Prediction of postoperative liver regeneration from clinical information using a data-led mathematical model. *Sci. Rep.* **6**, 34214; doi: 10.1038/srep34214 (2016).

## Supplementary Material

Supplementary Information

Supplementary Dataset 1

## Figures and Tables

**Table 1:** Summary of a training cohort (n = 157).

Factor	Mean	Range
Age (years)	68	28–88
Sex (male/female)	124/33	
Diabetes mellitus (yes/no)	48/109	
BMI (kg/m^2^)	22.8	13.9–32.9
Total bilirubin (mg/dL)	0.7	0.2–4.6
Albumin (g/dL)	3.8	2–4.9
AST(IU/L)	41.8	10–158
ALT (IU/L)	39.6	7–174
Prothrombin time (%)	102	50–150
Platelets (×10^3^/mm^3^)	19	4.2–49.1
ICGR15 (%)	14.6	2.3–72.2
Child-Pugh score (A/B)	147/10	
Hepatitis virus status		
HBV (yes/no)	58/99	
HCV (yes/no)	44/113	
Underlying disease		
HCC	91	
CCC	7	
HCC/CCC	1	
Other malignancy	2	
Metastatic liver tumor	53	
Benign liver disease	3	
Blood loss (g)	604	0–8280
Resected liver volume (g)	251	5–1380
Operation time (min)	263	50–798
Biliary complication (yes/no)	17/140	
Ascites (yes/no)	19/138	

*Abbreviations*: BMI = body mass index; AST = aspartate aminotransferase; ALT = alanine aminotransferase; ICGR15 = indocyanine green retention rate at 15 min; HCV = hepatitis C virus; HBV = hepatitis B virus; HCC = hepatocellular carcinoma; CCC = cholangiocellular carcinoma. Benign liver disease included liver stone and benign liver tumor. Other cancer included hemangiosarcoma and intrahepatic bile duct cystadenocarcinoma.

**Table 2:** Discriminant analyses of liver regeneration in a training cohort (n = 103).

	*K* or *M*	Predicted patient number	Matched patient number	Total predictive accuracy
(i) Preoperative	*K*	94	81	
	*M*	9	8	
	Total	103	89	89/103 (86.4%)
(ii) Perioperative	*K*	93	82	
	*M*	10	10	
	Total	103	92	92/103 (89.3%)
**Leave one out cross validation**				
(i) Preoperative	*K*	93	80	
	*M*	10	8	
	Total	103	88	88/103 (85.4%)
(ii) Perioperative	*K*	93	81	
	*M*	10	9	
	Total	103	90	90/103 (87.4%)

Accuracies of the predictions by linear discriminant functions (Eqs (4) and (5)) with (i) preoperative and (ii) perioperative factors.

**Table 3:** Discriminant analyses of liver regeneration in a validation cohort (n = 39).

	*K* or *M*	Predicted patient number	Matched patient number	Total predictive accuracy
(i) Preoperative	***K***	36	31	
	***M***	3	2	
	**Total**	39	33	33/39 (84.6%)
(ii) Perioperative	***K***	35	31	
	***M***	4	3	
	**Total**	39	34	34/39 (87.2%)

Accuracies of the predictions by linear discriminant functions (Eqs (4) and (5)) with (i) preoperative and (ii) perioperative factors.
