# Supplementary material for: Prediction of postoperative liver regeneration from clinical information using a data-led mathematical model
Source: Sci Rep. 2016 Oct 3;6:34214. doi: 10.1038/srep34214 (PMC5046126; doi:10.1038/srep34214)
Supplement: Supplementary Information [file srep34214-s1.pdf]

SUPPLEMENTAL INFORMATION

Prediction of postoperative liver regeneration from clinical information using a data-led mathematical model

Kimiyo N. Yamamoto[1,2,*], Masatsugu Ishii[1,*], Yoshihiro Inoue[1,†], Fumitoshi Hirokawa[1], Ben D. MacArthur[3,4], Akira Nakamura[5], Hiroshi Haeno[2], and Kazuhisa Uchiyama[1].

[1]Departments of General and Gastroenterological Surgery, Osaka Medical College Hospital, Osaka, Japan.

[2]Mathematical Biology Laboratory, Department of Biology, Faculty of Sciences, Kyushu University, Fukuoka, Japan.

[3]Mathematical Sciences, University of Southampton, SO17 1BJ, UK.

[4]Human Development and Health, Faculty of Medicine, University of Southampton, SO17 1BJ, UK.

[5]Department of Radiation Oncology, Massachusetts General Hospital, Boston, MA, USA.

*These authors contributed equally to this work.

† Corresponding author.

Table of Contents

S1. The basic mathematical model page 3

S2. Supplementary Tables pages 4-13

S3. Supplementary Figures pages 14-20

S1. The basic mathematical model

In this section, let us provide the derivation of the solution of equation (1) in main text. In the model, resected liver volume converges to either of two states, *K* and *M*: *K* represents the original volume of the liver in each patient before surgery, while *M* represents the reduced volume. Then, liver volume after surgical resection, *y(t)*, is given by

$$\frac{dy(t)}{dt} = r(\frac{K - y(t)}{K})(\frac{y(t) - M}{M}) \tag{S1}$$

Here, *r* denotes the regeneration rate per day. From equation (1), we have

$$(\frac{1}{y(t) - M} + \frac{1}{K - y(t)})dy(t) = r(K - M)dt \tag{S2}$$

By integrating both sides of the equation (S2), we have

$$\ln\left|\frac{y(t) - M}{K - y(t)}\right| = r(K - M)t + C \tag{S3}$$

Finally, the analytical solution of equation (S1) is given by

$$y(t) = \frac{CKe^{r(K-M)t} + M}{1 + Ce^{r(K-M)t}} \tag{S4}$$

S2. Supplementary Tables.

Please see Dataset 1 for Supplementary Tables S1 and S2.

Supplementary Table 3. Summary of the clinical cohort (validation cohort, n=39).

Factor	Mean	Range
Age (years)	66	33-85
Sex (male/female)	27/12	
Diabetes mellitus (yes/no)	6/33	
BMI (kg/m^2)	23.0	18.3-28.7
Total bilirubin (mg/dL)	0.7	0.2-2.7
Albumin (g/dL)	3.9	2.9-4.8
AST(IU/L)	34.9	15-68
ALT (IU/L)	31.6	10-78
Prothrombin time (%)	103	49-146
Platelets ($\times 10^3/mm^3$)	21.6	6-52.5
ICGR15 (%)	11.1	1.5-33.8
Child-Pugh score (A/B)	39/0	
Hepatitis virus status		
HBV (yes/no)	5/34	
HCV (yes/no)	7/32	
Underlying disease		
HCC	15	
CCC	7	
HCC/CCC	0	
Other Malignancy	2	
Metastatic liver tumor	15	
Benign liver disease	0	
Blood loss (g)	938	0-7270
Resected liver volume (g)	405	10-1139
Operation time (min)	300	115-810
Biliary complication (yes/no)	4/35	
Ascites (yes/no)	9/30	

Supplementary Table 4. Univariate analysis of clinical variables for regeneration volume at 5 months after surgery in the subset of patients who decreased in size and showed regeneration less than 90% of their original liver volumes (n=15).

Factor	Coefficient	*P*	
Age	2.1×10^{-3}	0.470	
Sex	-2.0×10^{-2}	0.733	
Diabetes mellitus	-4.7×10^{-2}	0.411	
Total bilirubin	3.1×10^{-2}	0.658	
Albumin	-2.3×10^{-2}	0.694	
AST	-1.5×10^{-3}	0.0930	
ALT	-1.6×10^{-3}	0.0774	
PT	-6.2×10^{-4}	0.693	
PLT	1.1×10^{-3}	0.642	
ICGR15	-3.4×10^{-4}	0.806	
Child-Pugh score	9.4×10^{-2}	0.299	
BMI	8.1×10^{-3}	0.270	
Blood loss	-1.3×10^{-4}	0.0149	*
Pre-ope liver volume	-1.7×10^{-4}	0.0219	*
Operation time	-1.9×10^{-6}	0.991	
Ascites	-1.0×10^{-2}	0.852	
Biliary	2.0×10^{-2}	0.827	
HBV	2.4×10^{-2}	0.621	
HCV	-7.0×10^{-3}	0.886	
Resection level	-8.0×10^{-2}	0.673	

Abbreviations: Blood loss = intraoperative blood loss; Biliary = postoperative biliary complication

*Statistically significant

Supplementary Table 5. Correlation coefficients for paired clinical information in the subset of patients who decreased in size and showed regeneration less than 90% of their original liver volume (n=15).

	Age	Sex	DM	T-bil	Albumin	AST	ALT	PT	PLT	ICGR15	BMI	Liver volume	Blood loss	Op. time	Ascites	Biliary	HBV	HCV	Resect
Age	1																		
Sex	-0.147	1																	
DM	-0.048	0.25	1																
T-bil	-0.37	-0.445	-0.364	1															
Albumin	-0.099	0.484	-0.359	-0.419	1														
AST	0.281	-0.305	-0.134	0.261	-0.256	1													
ALT	0.327	-0.286	-0.292	0.046	0.093	0.807*	1												
PT	0.305	0.547	0.418	-0.819*	0.354	-0.289	-0.296	1											
PLT	0.096	0.624	0.079	-0.607	0.615	-0.484	-0.402	0.782	1										
ICGR15	-0.198	-0.497	0.111	0.764	-0.817*	0.225	0.051	-0.65	-0.567	1									
BMI	-0.079	-0.134	-0.535	0.005	0.392	-0.444	-0.054	-0.084	0.183	NA	1								
Liver volume	0.094	-0.538	0.028	0.366	-0.504	-0.312	-0.311	-0.296	-0.197	0.662	0.163	1							
Blood loss	-0.021	0.339	0.34	-0.24	0.077	0.095	-0.049	0.496	0.348	-0.001	-0.08	-0.136	1						
Op. time	-0.244	-0.149	-0.189	-0.103	0.241	0.241	0.229	0.008	0.053	-0.158	-0.066	-0.103	0.328	1					
Ascites	-0.519	-0.105	-0.189	0.381	-0.104	0.235	-0.075	-0.373	-0.157	-0.001	-0.233	-0.263	-0.117	0.411	1				
Biliary	0.06	-0.452	0.075	0.034	-0.357	0.376	0.19	-0.304	-0.543	-0.17	-0.443	-0.123	-0.253	0.26	0.461	1			
HBV	0.426	-0.535	-0.134	-0.005	-0.258	0.465	0.396	-0.044	-0.341	0.011	0.071	-0.157	-0.074	-0.177	-0.05	0.443	1		
HCV	0.343	-0.354	-0.707	-0.029	0.33	-0.017	0.179	-0.106	0.142	-0.24	0.378	0.228	-0.148	0.34	-0.045	0.107	0.189	1	
Resect	0.3	0	0	0.072	-0.224	0.559	0.498	-0.125	-0.297	0.052	-0.378	-0.208	-0.133	-0.274	-0.124	0.213	0.378	-0.1	1

Abbreviations: DM = diabetes mellitus; T-bil = total bilirubin; Liver volume = pre-operative liver volume; Blood loss = intraoperative blood loss; Op.time = operation time; Biliary = postoperative biliary complication; Resect = resection level

*Statistically significant according to Pearson's correlation analyses.

Supplementary Table 6. Multivariate analysis of clinical variables for regeneration volume at 5 months after surgery in the subset of patients who decreased in size and showed regeneration less than 90% of their original liver volume (n=15).

Factor	Coefficient	*P*	
Liver Volume	-1.29×10^{-1}	0.0411	*
Blood loss	-9.91×10^{-2}	0.0596	.
Intercept	1089.93	< 0.001	*

Abbreviations: Liver volume = pre-operative liver volume; Blood loss = intraoperative blood loss
* Statistically significant

Supplementary Table 7. Univariate analysis of prognostic factors for regeneration rate *r* (n=103).

Factor	Coefficient	*P*	
Age	4.1×10^{-3}	0.38	
Sex	-3.9×10^{-2}	0.69	
Diabetes mellitus	1.0×10^{-1}	0.22	
Total bilirubin	-9.1×10^{-2}	0.21	
Albumin	-1.9×10^{-1}	0.026	*
AST	-4.3×10^{-3}	0.010	*
ALT	-3.5×10^{-3}	0.014	*
PLT	9.1×10^{-3}	0.079	.
ICGR15	-8.9×10^{-3}	0.037	*
Child-Pugh score	3.1×10^{-1}	0.14	
BMI	-9.2×10^{-3}	0.42	
Pre-ope liver volume	-4.6×10^{-4}	0.00046	*
Blood loss	-9.2×10^{-5}	0.0075	*
Operation time	-1.9×10^{-4}	0.54	
Biliary	-1.8×10^{-1}	0.13	
Ascites	-2.0×10^{-1}	0.085	.
HBV	-1.8×10^{-1}	0.023	*
HCV	-1.8×10^{-1}	0.037	*
Resection level	5.8×10^{-1}	0.0057	*

Abbreviations: Blood loss = intraoperative blood loss; Biliary = postoperative biliary complication

*Statistically significant

Supplementary Table 8. Correlation coefficients for paired clinical information (n=103).

	Age	Sex	DM	T-bil	Alb	AST	ALT	PLT	ICGR15	Child	BMI	Liver volume	Blood loss	Op. time	Ascites	Biliary	HBV	HCV	Resect
Age	1																		
Sex	-0.071	1																	
DM	0.152	0.047	1																
T-bil	-0.123	0.09	0.018	1															
Alb	-0.14	0.016	-0.055	-0.262	1														
AST	0.151	-0.076	0.092	0.236	-0.25	1													
ALT	-0.015	0.007	0.082	0.207	-0.11	0.823*	1												
PLT	-0.261	0.159	-0.173	0.074	-0.052	-0.152	-0.19	1											
ICGR15	0.073	-0.107	0.037	0.205	-0.277	0.297	0.196	-0.376	1										
Child	-0.077	-0.108	-0.197	-0.483	0.338	-0.184	-0.159	-0.039	-0.119	1									
BMI	-0.047	0.063	0.078	-0.109	0.253	-0.029	-0.026	-0.174	0.285	0.043	1								
Liver volume	-0.335	0.325	-0.061	0.106	-0.048	0.175	0.164	0.154	-0.049	-0.089	0.248	1							
Blood loss	0.118	0.144	-0.047	0.192	-0.171	0.141	-0.005	0.103	-0.018	-0.244	-0.049	0.229	1						
Operation time	-0.194	0.097	-0.114	0.19	-0.053	-0.037	-0.06	0.368	-0.161	-0.122	0.062	0.266	0.484	1					
Ascites	-0.079	-0.007	-0.058	0.252	-0.198	0.193	0.063	-0.089	0.073	-0.075	-0.024	0.17	0.393	0.36	1				
Biliary	0.082	0.009	0.11	0.047	-0.158	0.158	0.059	0.065	-0.103	-0.067	0.041	0.213	0.286	0.329	0.19	1			
HBV	-0.14	-0.175	-0.079	0.125	0.127	0.125	0.066	-0.157	0.068	0.079	0.157	0.056	-0.03	-0.174	0.129	-0.128	1		
HCV	0.164	-0.169	0.185	-0.089	-0.045	0.256	0.27	-0.473	0.222	0.018	-0.028	-0.072	-0.041	-0.314	0.078	-0.192	0.112	1	
Resect	-0.08	0.137	-0.18	0.111	-0.097	-0.036	-0.138	0.483	-0.233	-0.023	-0.169	0.135	0.323	0.419	0.288	0.155	-0.222	-0.339	1

Abbreviations: DM = diabetes mellitus; T-bil = total bilirubin; Alb = albumin; Child = Child-Pugh score; Liver volume = pre-operative liver volume; Blood loss = intraoperative blood loss; Biliary = postoperative biliary complication; Resect = resection level

*Statistically significant according to Pearson's correlation analyses.

Supplementary Table 9. Multivariate analysis of prognostic factors for regeneration rate *r* (n=103).

Factor	Coefficient	*P*	
Albumin	1.51×10^{-1}	0.051	.
ALT	-1.5×10^{-3}	0.24	
ICGR15	-4.7×10^{-3}	0.22	
Liver Volume	-4.0×10^{-4}	0.0011	*
Blood loss	-1.1×10^{-4}	0.00088	*
HBV	-1.0×10^{-1}	0.15	
HCV	-5.8×10^{-2}	0.48	
Resection level	6.9×10^{-1}	0.0012	*

Abbreviations: Liver volume = pre-operative liver volume; Blood loss = intraoperative blood loss

* Statistically significant.

Supplementary Table 10. Comparison of the number of patients who were predicted to proceed in the positive or negative direction by using initial points versus overall time series points (n=123).

	Overall time points	Initial points	
K	99	*K*	83
		M	16
M	24	*K*	7
		M	17

Supplementary Table 11. Discriminant analyses of liver regeneration in a training cohort without cases with unresponsive regeneration response postoperatively (n=89).

	K or *M*	Predicted patient number	Matched patient number	Total predictive accuracy
(i) Preoperative	*K*	79	70	
	M	10	8	
	Total	89	78	78/89 (87.6%)
(ii) Perioperative	*K*	79	71	
	M	10	9	
	Total	89	80	80/89 (89.8%)
Leave one out cross validation				
	K or *M*	Predicted patient number	Matched patient number	Total predictive accuracy
(i) Preoperative	*K*	79	69	
	M	10	7	
	Total	89	76	76/89 (85.4%)
(ii) Perioperative	*K*	78	70	
	M	11	9	
	Total	89	79	79/89 (88.8%)

Accuracies of the predictions by linear discriminant functions (Eqs. (4) and (5)) with (i) preoperative and (ii) perioperative factors.

S3. Supplementary Figures.

Supplementary Figure 1. The framework of the study.

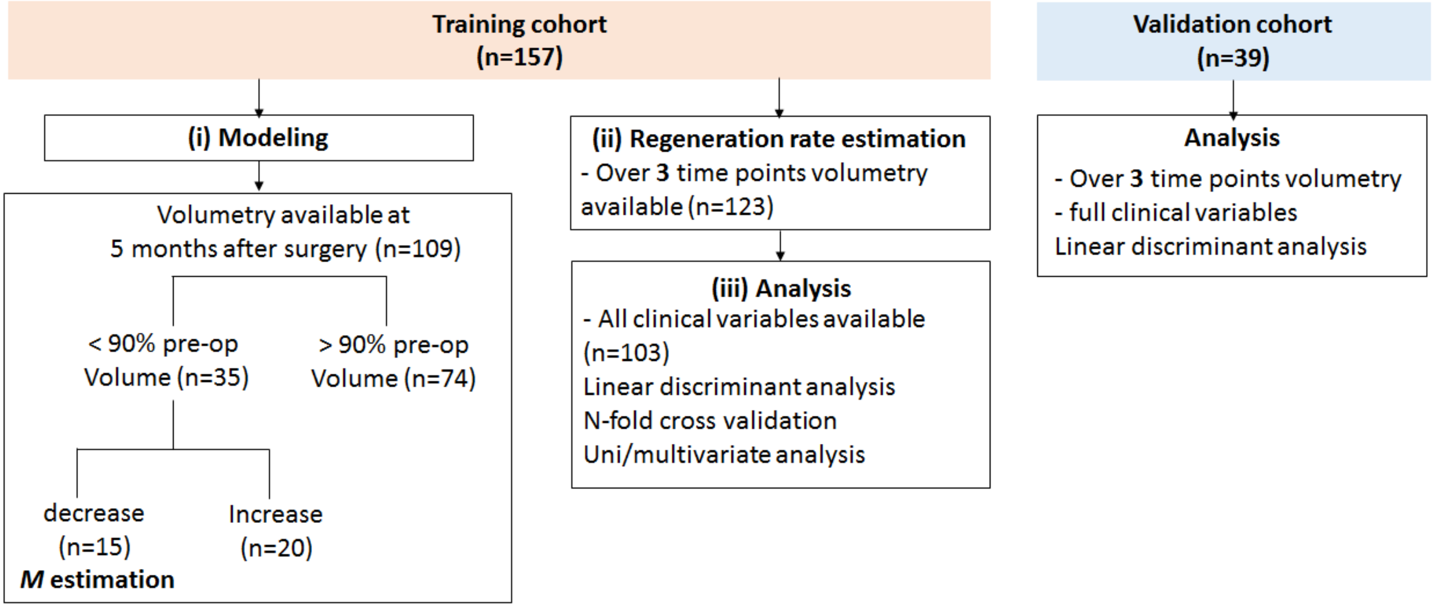

A training cohort consisting of 157 patients were used for (i) modeling, (ii) regeneration rate estimation, and (iii) analysis in the study. In modeling, 15 patients out of 109 patients who have liver volumetry at five months after surgery were analyzed to define *M*. 123 patients out of 157 patients had more than three time-series post-operative liver volume information, and were estimated their regeneration rates by non-linear least-squares curve-fitting. Of 123 patients, 103 patients with full clinical variables were enrolled in linear discrimination analysis with or without cross validation. A validation cohort consisting of 39 patients, who had more than three time points volumetry and full clinical variables were used to test the accuracy of the model.

Supplementary Figure 2. Distribution of estimated regeneration rates.

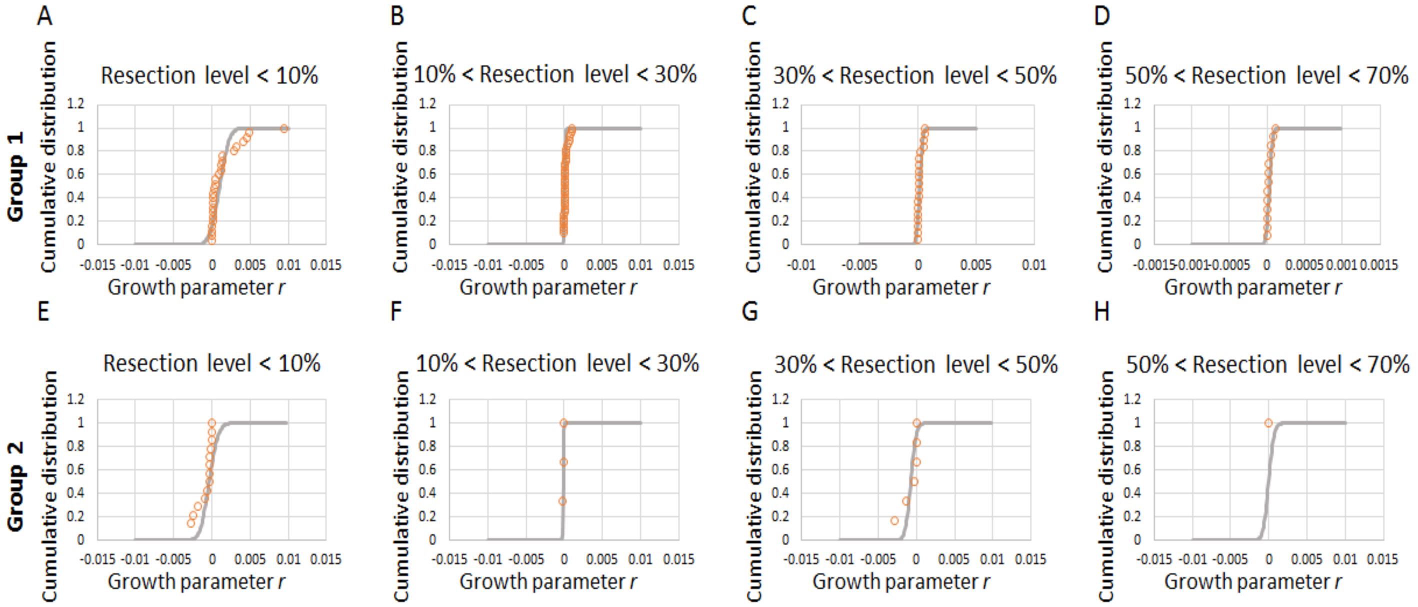

Fitting of estimated regeneration rates to cumulative normal distribution are shown. Cases are subdivided into two groups: those in whom liver regained its original size (Group 1) (panels A-D); and those who experienced a reduction in liver size (Group 2) (panels E-H). Both groups are then categorized into four subgroups according to resection levels: $\varepsilon = \frac{y_2}{y_3}$ (Fig. 2B): i) $\varepsilon < 0.1$; ii) $0.1 \leq \varepsilon < 0.3$; iii) $0.3 \leq \varepsilon < 0.5$; and iv) $0.5 \leq \varepsilon < 0.7$. Red circles and gray lines represent estimated regeneration rates and fitted normal distribution. Parameters are N(0.0015,0.0022), N(0.00042,0.0007), N(00001,0.0002), N(0.000033,0.000035), N(-0.0033,0.0095), N(-0.0001,0.0001), N(-0.00075,0.0012), and N(-0.00001,0.001) in A, B, C, D, E, F, G, and H, respectively. $N(\mu, \sigma)$ represents a normal distribution with mean μ and variance σ.

Supplementary Figure 3. Representative trajectories proceeding further in the positive or negative direction that are not governed by the initial direction.

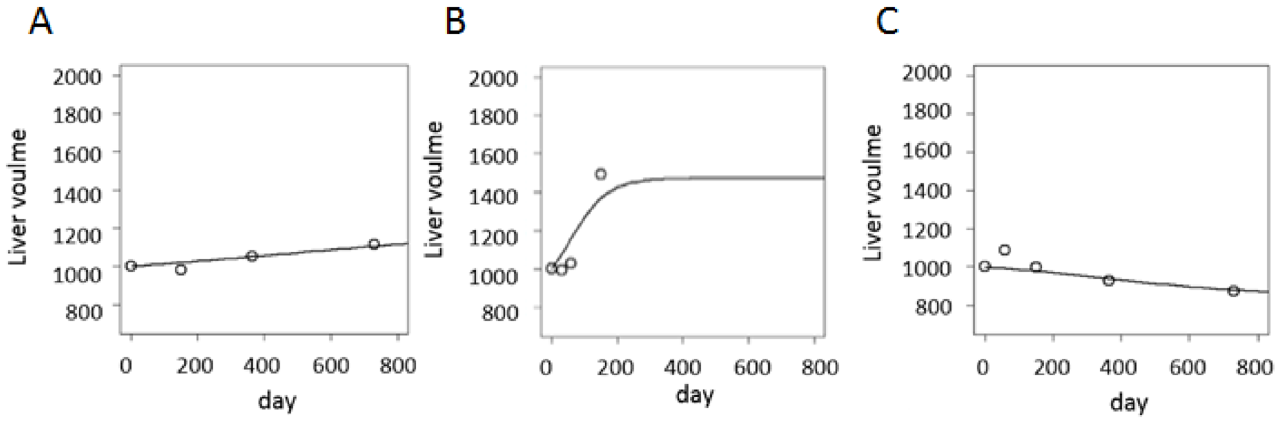

A and B) Trajectories in cases whose regeneration rates were estimated to be positive despite the initial decrease in their liver volumes but the increase at later time (ID=24 and ID=125 in Table S1). C) A trajectory in case whose regeneration rate was estimated to be negative despite the initial favorable recovery in liver volume but a decrease at later time (ID=22 in Table S1).

Supplementary Figure 4. Kaplan-Meier curves for overall survival.

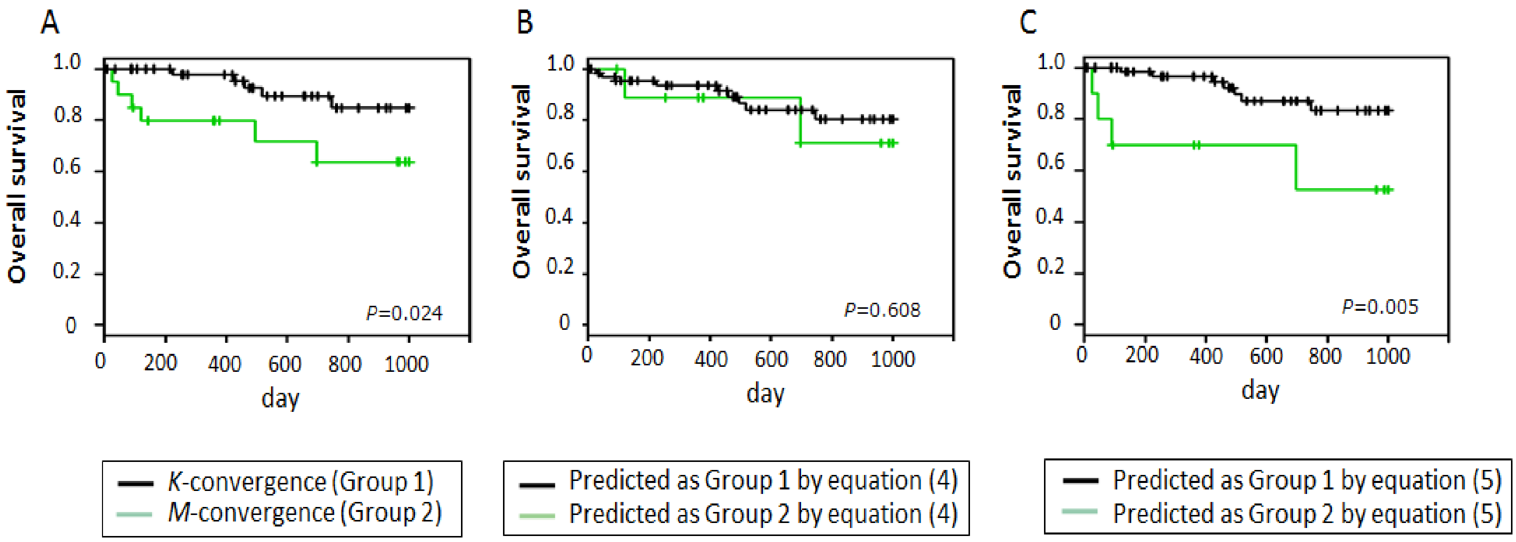

A) Overall survivals in the groups of patients converging to *K* (Group 1) or *M* (Group 2) as determined by curve-fitting of equation (2) to clinical data with the nonlinear least-squares method (n=76). B and C) Overall survival in the two groups of patients predicted to show *K* (Group 1) or *M* (Group 2) by discriminant analysis of regeneration rate *r* using (B) preoperative (equation (4)) and (C) perioperative (equation (5)) clinical factors.

Supplementary Figure 5. Kaplan-Meier curves for overall survival.

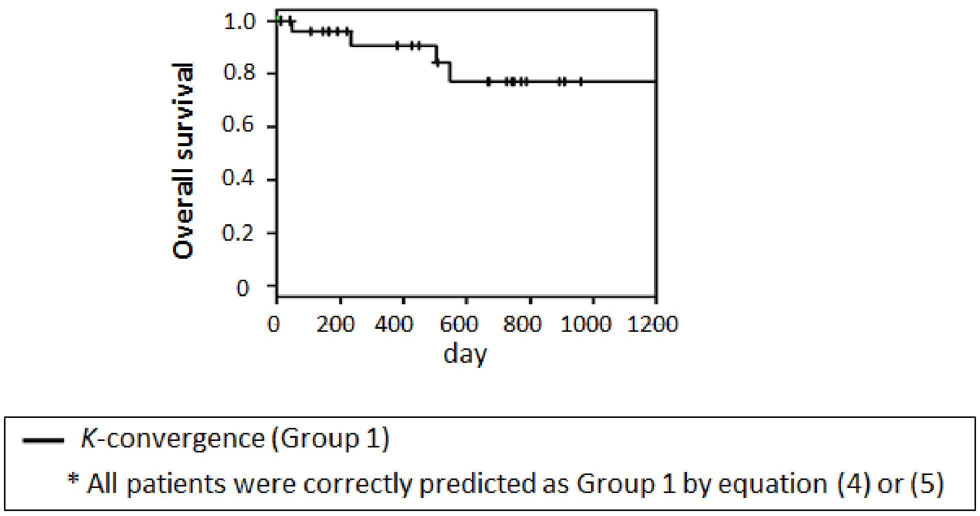

Overall survivals in patients with metastatic disease. All patients converged to *K* by curve-fitting of equation (2) to clinical data with the nonlinear least-squares method. All patients were predicted to converge *K* by both equation (4) and (5) (n=27).

Supplementary Figure 6. Schematic illustration of clinical application.

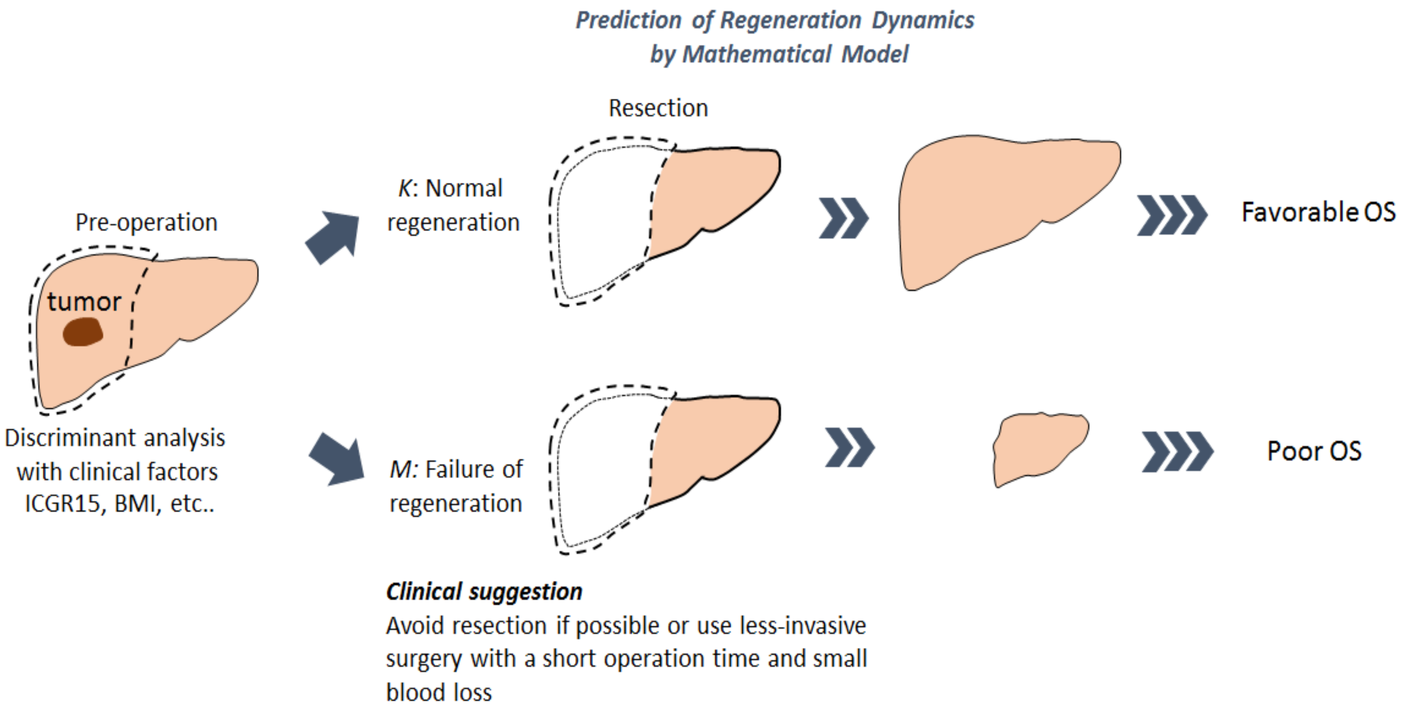

Liver regeneration can be predicted by pre- and perioperative clinical factors. If the solution of the discriminant equation is positive, the resected liver is predicted to recover to the original size. If the solution is negative, the liver is expected to show a reduction in size. In this situation, patients may avoid resection if possible, or may receive minimally invasive surgery so that the operation time is shortened and intraoperative blood loss and postoperative complication are reduced.

Supplementary Figure 7.

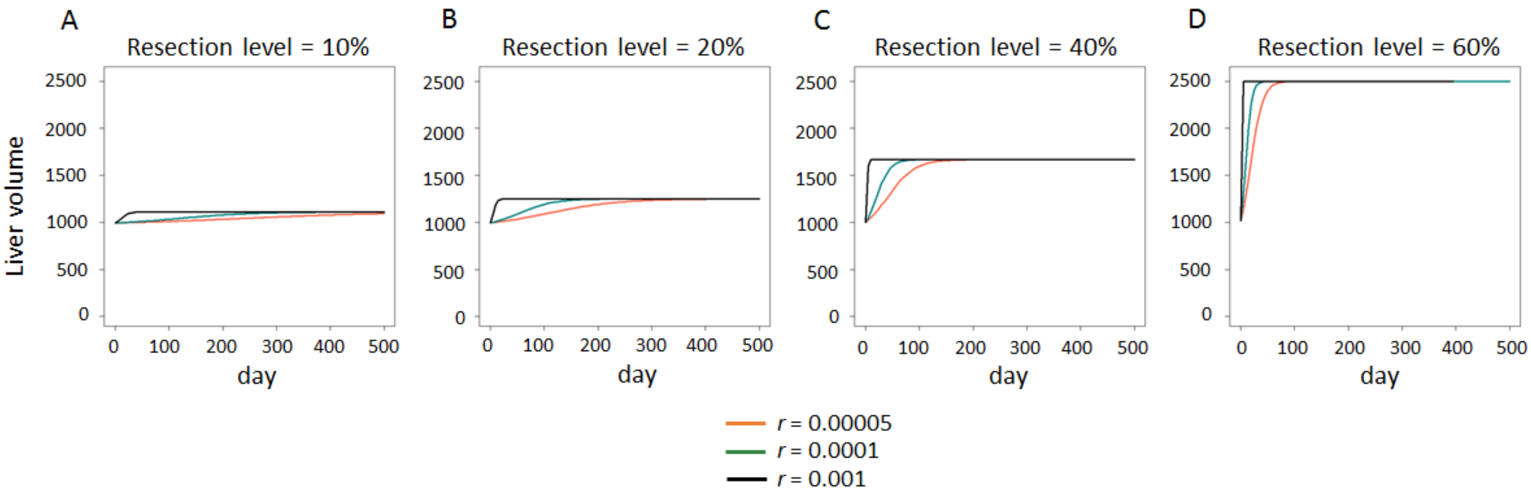

Panels represent regeneration dynamics of cases with different positive regeneration rates *r* in accordance with four resection levels; (A) 10%, (B) 20%, (C) 40%, and (D) 60% based on the equation (2).
